# Academic competence and special educational needs as outcomes of early onset epilepsy: A population-based prospective follow-up study

**DOI:** 10.1016/j.ebr.2025.100777

**Published:** 2025-04-30

**Authors:** Kati Rantanen, Jenna Mäntylä, Eeva Kettunen, Annina Saunajoki, Kai Eriksson

**Affiliations:** a Department of Rehabilitation and Psychosocial Support, Tampere University Hospital, Wellbeing Services County of Pirkanmaa, Finland; b Department of Psychology, Faculty of Social Sciences, Tampere University, Finland; c Department of Psychology, Faculty of Medicine, University of Helsinki, Finland; d Tampere Center for Child, Adolescent and Maternal Health Research (TamCAM) and Faculty of Medicine and Health Technology, Tampere University, Finland; e Department of Pediatric Neurology, Tampere University Hospital, Wellbeing Services County of Pirkanmaa, Finland

**Keywords:** Complicated epilepsy, Uncomplicated epilepsy, Academic competence, Special educational needs, Intellectual disability

## Abstract

Epilepsy in children is associated with significant academic challenges, particularly among those with early-onset seizures and comorbid intellectual disabilities (ID). This study aims to bridge gaps in existing research by examining long-term academic outcomes and special educational needs (SEN) in a cohort of children with early-onset epilepsy, including those with complicated epilepsy (CE) and major comorbidities. The study followed a population-based cohort of 64 children with epilepsy (CWE) aged 3–6 years, identified from the Pediatric Neurology Unit at Tampere University Hospital. Six years later, 43 children (67 %) participated in the follow-up. Academic competence was assessed using the Teacher Report Form (TRF), which also provided data on SEN. The findings revealed that 67 % of participants had low academic competence, with a significant proportion requiring special educational support. Children with CE had notably lower academic performance compared to those with uncomplicated epilepsy (UE). The need for SEN was higher among children with CE (81 %) than those with UE (24 %), with epilepsy type emerging as a significant predictor for SEN. Despite the small sample size and limitations in generalizability, the study underscores the pervasive impact of early-onset epilepsy on academic outcomes and highlights the necessity for ongoing educational support. Future research should focus on larger cohorts and explore the efficacy of tailored educational interventions to improve the academic performance and quality of life for CWE. Regular screening and collaboration with educators are essential to address the unique needs of these children effectively.

## Highlights

• Children with epilepsy (CWE) face significant academic challenges.
• About 67 % of children with early onset epilepsy show low academic competence.
• Those with complicated epilepsy (CE) and intellectual disability are most affected.
• 81 % of children with CE need special education, vs 24 % with uncomplicated epilepsy.
• The persistency of academic difficulties in early onset epilepsy is highlighted.

## 1 Introduction

Epilepsy is a prevalent neurological condition that affects children worldwide, often with significant implications for their cognitive function and learning skills [1]. Children with epilepsy (CWE) face an elevated risk of academic difficulties and often require special educational support. Systematic reviews [2,3] and large cohort studies [4] consistently show lower academic competence in CWE compared to healthy peers, other chronic conditions, or normative data, both in standardized objective measures of academic achievement and subjective teacher reports of academic competence [2]. However, there are gaps in the earlier studies, as they have mainly focused on school-aged children representing epilepsies with later seizure onset and excluded children with additional neurological and/or neurobehavioral comorbidities, such as intellectual disability (ID) [2].

Several factors contribute to academic challenges faced by CWE. These include the underlying cause of epilepsy, age at seizure onset, the presence of active epilepsy or persistent seizures, and antiepileptic medication (AED) [[4], [5], [6]]. Neurobehavioral comorbidities, including ID and ADHD, are common in CWE [7] often exacerbate academic difficulties [5]. These comorbidities are particularly high among children with complicated epilepsy (CE) i.e. those with underlying structural / metabolic etiology or other central nervous system pathology or additional neurologic conditions or IQ < 70–80 but are also elevated in those with uncomplicated epilepsy (UE, i.e. with no other neurological signs or symptoms and intellectual function within the normal range) [1,7]. Most studies on academic problems have typically focused on epilepsies that have onset at school-age (e.g. [8]), despite the fact that half of childhood epilepsies begin at early age before entering school [9].

Epidemiological population and community based studies in CWE demonstrate high rates of ID (ranging from 20-40 %) [[10], [11], [12]] and psychiatric comorbidities (ranging from 35 to 50 %, [13]), with even higher in those with additional impairments. Neurobehavioral problems are encountered in almost two-thirds of children with early onset epilepsy [14], and a risk for ID is elevated [15]. Particularly unfavorable long-term neurobehavioral and learning outcomes are related to seizures with the onset before age of 5 or 6 [10,[15], [16], [17]]. In addition, psychiatric comorbidities or behavioural disturbances further contribute to learning problems along with underlying neurocognitive deficits [6].

Age at onset of seizures is a predictive factor for learning achievement at school-age, and younger age at seizure onset is associated with lower academic achievement also after controlling for IQ [5]. Recently, Dreier et al. [4] demonstrated in a large Danish registry-based nation-wide matched cohort study that poor school performance was evident in subgroups of children with early (<3 years) and later (≥ 3 years) onset CE and UE. Poorer academic performance compared to controls in standardized tests in language and mathematics were reported in all seizure-onset subgroups, also those with UE, emphasizing a widespread need of educational support in school-aged CWE [4].

Academic difficulties in children with active epilepsy can also partly be attributed to lowered global cognition [5], although children with well-controlled seizures face challenges. Studies have reported that a significant percentage (over 30 %) of children with active epilepsy attend specialized schools and have special educational needs (SEN) statements [5,18]. The link between seizure-related variables and academic achievement is inconsistent [15], with most studies focusing on active epilepsy [19] and showing a strong association with learning difficulties [5]. Academic achievement difficulties seem to be quite stable over time even among those CWE with improved seizure frequency and UE [2].

An increased need for additional learning support and special educational services reflects persistent nature of learning difficulties in CWE. Studies have shown that the need for special educational services remains significantly higher in CWE compared to controls after several years post-diagnosis [20,21]. This highlights the persistent and pervasive nature of academic difficulties in CWE, necessitating ongoing support and intervention.

Prospective follow-up studies are still needed to understand academic competence and the long-term academic trajectories of children with early-onset seizures. Previous studies on the impact of epilepsy on children's academic competence and SEN outcomes have usually focused on later onset epilepsies and excluded children with CE or major comorbidities (see e.g. review by Wo et al. [2]. This exclusion has created a significant gap in the research, as it fails to encompass the full spectrum of children affected by early-onset seizures. Consequently, there is an evident need for more comprehensive long-term studies that do not impose such exclusion criteria. This prospective follow-up study aims to bridge this gap by examining the long-term outcomes in academic competence in a cohort of children with early-onset epilepsy, and including also those with major comorbidities related to CE. By doing so, it aims to describe the frequency of special needs support required for these children and to provide deeper insight into the epilepsy related factors contributing to their academic competence and SEN.

## 2 Material and methods

### 2.1 Participants

A population-based cohort of 64 CWE aged 3 to 6 years that was first identified from the Paediatric Neurology Unit at Tampere University Hospital Pediatric Neurology Unit, Tampere University Hospital, Finland, which is the only center for pediatric neurology services in the hospital district [12]. At the time of the study, the total population of the hospital district was 464,976, of whom 19,821 were children aged 3–6 years 11 months. Data for this follow-up study interval was 6 years. At the follow-up, six children (9.4 %) of the original cohort had died, and most of them (n = 5) had complicated epilepsy (CE) i.e. with underlying central nervous system pathology, IQ < 70 or additional neurologic conditions. Of the remaining 58 CWE, 43 (74.1 %) children participated in this study. Among the 15 non-participants one participant had moved and could not be reached, another was severely impaired and institutionalized, and the remaining 13 participants declined to participate. Although specific reasons for refusal were not required, some guardians voluntarily provided explanations. The most common reasons cited were busy schedules and time constraints.

Medical data and results of earlier neuropsychological assessment were reviewed from children’s medical and psychological records. The children’s background variables included sex, age, diagnosis, medication, and preschool aged IQ assessed with standardized psychological tests (i.e. Wecshler Scales of Intelligence (Wechsler, 1999, 1995), or Bayley Scales for Infant Development-II; Bayley 1994). For those children unable to participate in standardized psychological assessment (e.g. due to severe mental impairment), the level of cognitive functioning was determined from medical and psychological records, parental reports, and, when available, observations from daycare. No psychological assessment data was available for three participants. Demographic and background data for the participants is presented at Table 1. At follow-up, participants (20 boys, 23 girls) were 9–14 years (mean 11.71). Mean age at the onset of epilepsy was 2.2 years and mean duration of epilepsy at follow-up was 8.9 years. Majority of children were still on antiseizure medication (ASM). Sixteen children encountered recurrent seizures (at least monthly), while 27 children had been seizure free for over 2 years. Seventeen (39.5 %) had uncomplicated epilepsy (UE) and 26 (60.5 %) CE. Statistically significant differences between the participants (n = 43) and nonparticipants (n = 15) were not found regarding UE/CE, ID, age at onset, recurrent seizures), indicating that participants were likely to represent a cohort of children with early onset epilepsies. At follow-up, 65 % participants had been diagnosed with comorbid neurodevelopmental or psychiatric disorder, most typically ID (39.5 %). Other comorbid neurodevelopmental disorders (e.g. mixed developmental disorder) were reported in 25.6 % of the participants.

**Table 1:** Demographic and background data for the participants.

	Study group at 6 years of follow-up
N	43
Sex (male/female)	20 / 23
Age, years: m (sd)	11.71 (1.27)
Age at onset, years	2.19 (1.63)
Duration, years	8.92 (1.97)
Etiology (3–6 v)	
Genetic	5
Symptomatic	16
Unknown	22
Seizure type (3–6 v)	
Focal	24
Generalized	17
Unclassified	2
Antiepileptic medication	
No ASM	9
ASM	34
Seizure frequency	
Recurrent (≥monthly) seizures	16
No seizures (>2 years)	27
Comorbid neurodevelopmental diagnosis	28
Intellectual disability (ID)	17
Mixed developmental disorder	7
Developmental language disorder	1
Autism spectrum disorder	2
ADHD	1

### 2.2 Measures of academic competence and special educational needs

At follow-up, academic competence was assessed with the Teacher Report Form (TRF) of the Achenbach System of Empirically Based Assessment (ASEBA) for school-age children [22]. The TRF provides information about special remedial school services (Items IV-V), repetition of grades (VI), and other school problems, which are combined with ratings of academic performance *(Items VII-VIII)* for scoring on a subscale. The TRF asks each teacher to rate a child's academic performance in each subject on 5-point scales ranging from 1 = far below grade to 5 = far above grade. Scores for all the competence items are summed to provide a Total Academic Competence score. The raw scores were individually converted to T scores ranging from 35 to 65. T scores ≥ 40 were considered within normal range and cut-off score for low academic competence was T < 39 indicating borderline and clinical range academic difficulties [22]. The TRF form also includes space for the teacher's free comments on the child's school performance (including strengths and concerns). SEN was also determined by the TRF reports that include items of special education and remediation teaching received, and teachers more detailed description of what kind of support child is receiving and when.

### 2.3 Statistical analysis

IBM SPSS Statistics (version 27) software was used for the statistical analyses. Due to small sample size and distribution of variables non-parametric methods (Mann-Whitney, Wilcoxon signed ranked test) were used to group comparisons. Chi square test was used for categorical variables and Spearman’s rho for correlations. Due to missing replies, n varies in analyses between 41 and 43. Further, logistic regression analyses were carried out to assess the effect of UE vs CE, ASM usage, recurrent seizures and age at onset of seizures on the likelihood of SEN and academic performance. P-values < 0.05 were considered statistically significant. Spearman correlation approximately *p* ≥ 0.30 is interpreted as evidence for medium effect size.

### 2.4 Ethicall approval

This study was approved by the Ethics Committee of the Tampere University Hospital, Pirkanmaa Hospital District. Parents of CWE gave their informed written consent for participation.

## 3 Results

### 3.1 Academic competence and special learning support

At 6 year follow-up interval, participants attended comprehensive school in grades 2 through 8. Teachers report low academic competence (T score < 39) in 66.7 % of participants. This percentage includes 8 participants (18.6 %) who were attending special classes for children with severe ID, although their academic competence could not be formally assessed using the TRF. The Academic Performance was significantly lower in children with CE than in those with UE (*U* = 72.0, *p* = 0.019). Yet, teachers reported learning difficulties in reading and literature, mathematics and other core academic subjects (such as history, biology) in some of the children with UE (35 %). Academic competence in both groups was related to present seizure frequency (UE; *r* = 0.597, *p* < 0.05, and CE; *r* = 0.562, *p* = 0.023). Age at onset of seizures was not associated with academic competence in the UE or CE group, but preschool-age IQ was associated with academic performance in the UE group (*r* = 0.744).

Some special education teachers (n = 10) faced challenges in completing the TRF accurately, either due to difficulties following the completion instructions or by rating children based on individual goals outlined in their individualized educational plans (IEP) or using the special class as a reference group. This led to missing items and biased rating. Consequently, due to the small number of participants eligible for analysis, it was not feasible to use a logistic regression model to assess the effects of epilepsy-related variables on teacher-rated academic competence.

**Fig. 1:**
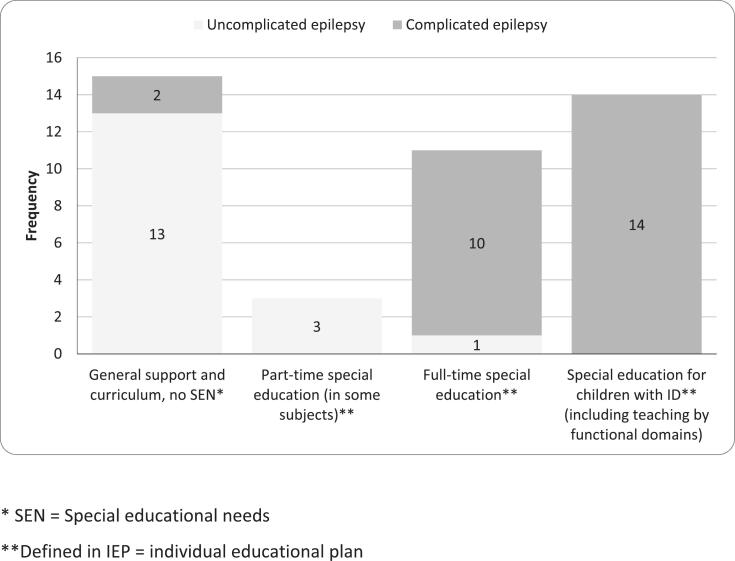
Frequency of special educational support in children with uncomplicated and complicated epilepsy. * SEN = Special educational needs. **Defined in IEP = individual educational plan.

In the cohort with early-onset epilepsy, 15 (35 %) attended mainstream education following the general curriculum, and required no special learning support (Fig. 1). However, occasional remedial teaching, small-group instruction and/or individual assistance may have been arranged as a part of general support. The majority of CWE (65 %) needed regular special educational support as defined in their IEP and 55.8 % required full-time special education. About 33 % of the participants was attending special education classes for children with ID, and some (n = 8) of them with the focus on teaching through functional domains (i.e. language and communication, cognitive skills, motor skills, social skills, activities of daily living) instead of academic subjects. The children with CE received special educational support (either part-time or full time) more frequently (81 vs. 24 %) than those with UE (*X*^2^(1) = 12.67, *p* = 0.001). Also, participants with recurrent seizures had SEN more likely than those without seizures (*X*^2^(1) = 7.71, p = 0.008). There were no differences between sex (*p* = 0.353), age at onset (*p* = 0.258), ASM usage (*p* = 0.647) and SEN.

### 3.2 Factors contributing to SEN

A logistic regression was used to assess the effect of UE vs CE, recurrent seizures and ASM on the likelihood of requiring SEN in CWE (Table 2). The overall model was statistically significant when compared to the null model (χ^2^(3) = 13.803, *p = 0*.003), and explained 39 % of the variation of SEN (Nagelkerke R^2^) and correctly predicted 78 % of cases. The group (UE/CE, *p* = 0.031) was the only significant predictor but recurrent seizures and AED usage were not.

**Table 2:** Binary logistic regression model with special educational needs (SEN) as outcome variable.

Variable	OR	95 % CI	*p*
Epilepsy group (UE - CE*)	6.165	1.181–32.181	0.031
Recurrent seizures (yes, no)	0.230	0.034–1.535	0.129
ASM usage	2.820	0.379–21.01	0.312

*UE = uncomplicated epilepsy, CE = Complicated epilepsy.

## 4 Discussion

This prospective follow-up study aimed to determine the long-term outcomes of early onset epilepsy on teacher-rated academic competence and SEN. The second aim was to examine epilepsy-related factors contributing to children's SEN and describe the nature of special educational support required by these children. The results demonstrated that teacher-rated academic competence was lower than expected for majority (67 %) of children with early onset epilepsy. This low academic competence reflects the inclusion of children with CE and comorbid ID, nearly all of whom received some form of special educational services. Similar findings regarding CE have been previously reported [6,21].

In addition to low academic competence, special educational support was required for 65 % of the cohort approximately 9 years after the onset of seizures. This is a slightly higher proportion of SEN than reported in earlier studies, which found SEN in 45–58 % of CWE 5–9 years post-diagnosis [5,21]. The need for special educational support was prevalent among CWE, with 65 % requiring regular special educational support as defined in their IEP. Epilepsy type (UE vs. CE) emerged as the only significant predictor for SEN, with pronounced difference in the proportion of children with CE and SEN compared to those with UE and SEN (81 % vs 24 %). The study found that the risk for SEN was over six times higher in children with CE compared to those with UE. However, also children with UE had special educational needs, although over half of these did not experience recurring seizures or were on antiepileptic drugs. This percentage of SEN was significantly lower than the 44 % reported for UE with low average and above IQ (>80) [21], but higher than the 13 % of pupils receiving enhanced or special support in Finnish comprehensive schools at the time of the study [23].

Interestingly, the age at onset of seizures was not a significant predictor in the limited age cohort of 3–6-year-old CWE. This result suggests the persistent learning difficulties in children with early onset epilepsy. Earlier, a population-based study reported that 34 % of children with active epilepsy attended specialized schools, and 64 % had SEN statements [5]. In Finland, special classes in regular comprehensive schools are intended for the pupils requiring intensive special education. According to the general principle, the learning support is systematically organized and gradually intensified based on pedagogical assessments (Basic Education Act, 628/1998, Amendment 642/2010). Every child is entitled to general learning support, but if this turns out to be insufficient, enhanced support or later special-needs support are offered based on a pedagogical statements and administrative decision. Given that most participants in this study received the most intensive, full-time, and long-term special learning support from specialized teachers, it can be concluded that milder forms of learning support (such as general or intensified support) within the Finnish school system would likely be insufficient to meet their needs.

The study highlights the academic challenges and special needs faced by CWE, particularly those with CE, who show markedly elevated rates of special education needs when compared to the general population. The disparity is striking, with 81 % of children with CE requiring special education services, in contrast to just 13 % in the general population. Furthermore, even children with UE experience a significantly higher rate of SEN, at 24 %. This highlights the substantial impact epilepsy can have on educational requirements and underscores the importance of targeted support and resources for these children. The findings based on teacher-rated academic competence indicate that a significant proportion of CWE struggle academically. This underscores the pervasive impact of early onset epilepsy on later educational outcomes. Effects can be pervasive and long-lasting as seen in a recent Australian study of young people (≤18 years) hospitalized with epilepsy. They were reported to have more than three times higher risk for not achieving national minimum standard for numeracy and reading compared to matched peers, and 27 % of them did not complete high school [24].

### 4.1 Limitations and Considerations

When interpreting the results, several limitations must be considered. The sample consists of children with early onset epilepsies (before school age), some of which represent severe and devasting epilepsies related to neurodegenerative conditions. Additionally, some epilepsy types, e.g. childhood absence epilepsies with more favorable cognitive and academic outcome are underrepresented due to their typically later seizure onset. However, to gain a comprehensive understanding of the full spectrum of children affected by early-onset seizures, it is essential to include children with CE and comorbid neuroimpairments. These children often require more intensive support and guidance, and for parents and caregivers additional neuroimpairments can be more impactful on daily life than the seizures themselves.

Despite using a population-based cohort, this study has limitations. The small sample size and inclusion of children with ID and additional neuroimpairments raise questions about the generalizability of the results to CWE with average intellectual abilities. The use of subjective teacher rating scales was used instead of objective academic achievement tests may lead to an overestimation of special educational needs in CWE. Alternatively, needs may also be underestimated due to teachers missing learning problems or attributing lower performance to epilepsy instead of learning difficulties. Including children with ID and additional neuroimpairments leads to a wide spectrum of children and considers those who need significant support in their daily life. However, focusing on early onset epilepsies excludes some epilepsy types, such as childhood absence epilepsy, which generally has a more favorable cognitive and academic outcome. Therefore, the results are not necessarily generalizable to all CWE. Nonetheless, we cannot disregard children with CE and comorbid neuroimpairments because they need more intensive support and guidance. For parents and caregivers, additional neuroimpairments may sometimes be more significant than seizures in coping with daily life.

Including a cohort of CWE with varying cognitive capacities results in compromises, such as using subjective teacher-reports instead of standardized tests of academic achievement, which may not be suitable or reliable for children with ID. The TRF used is not ideally suited to children with ID as seen in this study. Participants whose teaching was arranged by functional domains had to be excluded from the analysis of academic competence as there were inconsistencies in the completion of the TRF by special education teachers. These factors may have introduced bias and limited the generalizability of the findings. Moreover, the small sample size precluded the use of a logistic regression model to assess the effects of epilepsy-related variables on teacher-rated academic competence. Future studies with larger sample sizes and more rigorous data collection methods are needed to confirm these findings and provide a more comprehensive understanding of the academic challenges faced by CWE.

### 4.2 Implications and Future Directions

In conclusion, this study provides clinical insight into early onset epilepsies and the challenges faced by CWE, particularly those with CE. The findings highlight the significant academic challenges these children face and the necessity for tailored educational interventions and support. Regular screening is essential to recognize the needs for learning support in CWE. The study emphasizes the importance of early cognitive abilities in determining educational outcomes and calling for specific interventions to support these children in their academic pursuits. Schools and educators should recognize the potential academic difficulties of CWE and appropriate accommodations and support to ensure their success.

The significance of the findings lies in the early identification and provision of necessary learning supports, which are crucial for the development and learning outcomes of CWE, and especially those with comorbid ID. Early intervention can help mitigate the challenges these children face and enhance their educational experience. However, it remains unclear whether there is a gap in educational services for children with complicated versus uncomplicated seizures. While our study reports the frequency of services received, further research is needed to explore if specific gaps exist in the support provided to these subgroups. Understanding these gaps can help in refining educational strategies and ensuring that all children, regardless of the complexity of their seizure condition, receive appropriate and effective support.

Our findings also underscore the need for continued research into the adequacy of services for children with varying complexities of epilepsy. This will ensure that educational support is comprehensive and inclusive, catering to the diverse needs of all students. Further research should explore the long-term educational outcomes of CWE and the efficacy of different types of educational support. Understanding the unique needs of these children and developing tailored interventions will be crucial in improving their academic performance and overall quality of life. Collaboration with teachers are recommended as standard procedure as they may have limited knowledge or hold negative attitudes towards epilepsy [25].

## CRediT authorship contribution statement

**Kati Rantanen:** Writing – original draft, Supervision, Resources, Project administration, Methodology, Investigation, Funding acquisition, Formal analysis, Data curation, Conceptualization. **Jenna Mäntylä:** Writing – original draft, Visualization, Methodology, Investigation, Formal analysis, Data curation, Writing – review & editing. **Eeva Kettunen:** Writing – review & editing, Methodology, Investigation, Formal analysis. **Annina Saunajoki:** Writing – review & editing, Methodology, Investigation, Formal analysis. **Kai Eriksson:** Writing – review & editing, Supervision, Resources, Project administration, Investigation, Funding acquisition, Conceptualization.

## Funding

This follow-up study was financially supported by the State funding for university-level health research, Tampere University Hospital, Wellbeing services county of Pirkanmaa (Project No 9AA064) (KR, JM) and Arvo and Lea Ylppö Foundation (grant #202010016, KR). Open access funded by Helsinki University Library.

## Declaration of competing interest

The authors declare that they have no known competing financial interests or personal relationships that could have appeared to influence the work reported in this paper.

## References

[1] Nickels K.C., Zaccariello M.J., Hamiwka L.D., Wirrell E.C. (2016). Cognitive and neurodevelopmental comorbidities in paediatric epilepsy. Nat Rev Neurol.

[2] Wo S.W., Ong L.C., Low W.Y., Lai P.S.M. (2017). The impact of epilepsy on academic achievement in children with normal intelligence and without major comorbidities: A systematic review. Epilepsy Res.

[3] Reilly C., Neville B.G. (2011). Academic achievement in children with epilepsy: a review. Epilepsy Res.

[4] Dreier J.W., Trabjerg B.B., Plana-Ripoll O., Skipper N., Agerbo E., Cotsapas C. (2024). Epilepsy in childhood and school performance: A nation-wide cohort study. Brain: A J Neurol.

[5] Reilly C., Atkinson P., Das K.B., Chin R.F.C., Aylett S.E., Burch V. (2014). Academic achievement in school-aged children with active epilepsy: A population-based study. Epilepsia.

[6] Berg A.T., Caplan R., Baca C.B., Vickrey B.G. (2013). Adaptive behavior and later school achievement in children with early-onset epilepsy. Dev Med Child Neurol.

[7] Aaberg K.M., Bakken I.J., Lossius M.I., Lund Søraas C., Håberg S.E., Stoltenberg C. (2016). Comorbidity and childhood epilepsy: A nationwide registry study. Pediatrics.

[8] Dunn D.W., Johnson C.S., Perkins S.M., Fastenau P.S., Byars A.W., deGrauw T.J. (2010). Academic problems in children with seizures: Relationships with neuropsychological functioning and family variables during the 3 years after onset. Epilepsy Behav.

[9] Hunter M.B., Yoong M., Sumpter R.E., Verity K., Shetty J., McLellan A. (2020). Incidence of early-onset epilepsy: A prospective population-based study. Seizure.

[10] Berg A.T., Langfitt J.T., Testa F.M., Levy S.R., DiMario F., Westerveld M. (2008). Global cognitive function in children with epilepsy: A community-based study. Epilepsia (Series 4).

[11] Reilly C., Atkinson P., Das K.B., Chin R.F., Aylett S.E., Burch V. (2014). Neurobehavioral comorbidities in children with active epilepsy: a population-based study. Pediatrics.

[12] Rantanen K., Eriksson K., Nieminen P. (2011). Cognitive impairment in preschool children with epilepsy. Epilepsia.

[13] Besag F., Aldenkamp A., Caplan R., Dunn D.W., Gobbi G., Sillanpää M. (2016). Psychiatric and behavioural disorders in children with epilepsy: ILAE task force report: Epidemiology of psychiatric/behavioural disorder in children with epilepsy. Epileptic Disord.

[14] Hunter M.B., Yoong M., Sumpter R.E., Verity K., Shetty J., McLellan A. (2019). Neurobehavioral problems in children with early-onset epilepsy: A population-based study. Epilepsy Behav.

[15] Reilly C., Atkinson P., Memon A., Jones C., Dabydeen L., Das K.B. (2019). Global development and adaptive behaviour in children with early‐onset epilepsy:A population‐based case–control study. Dev Med Child Neurol.

[16] Sillanpää M. (2004). Learning disability: Occurrence and long-term consequences in childhood-onset epilepsy. Epilepsy Behav.

[17] Hunter R.M., Reilly C., Atkinson P., Das K.B., Gillberg C., Chin R.F. (2015). The health, education, and social care costs of school-aged children with active epilepsy: A population-based study. Epilepsia.

[18] Fleming M., Fitton C.A., Steiner M.F.C., McLay J.S., Clark D., King A. (2019). Educational and health outcomes of children and adolescents receiving antiepileptic medication: Scotland-wide record linkage study of 766 244 schoolchildren. BMC Public Health.

[19] Berg A.T., Langfitt J.T., Testa F.M., Levy S.R., DiMario F., Westerveld M. (2008). Residual cognitive effects of uncomplicated idiopathic and cryptogenic epilepsy. Epilepsy Behav.

[20] Oostrom K.J., Smeets-Schouten A., Kruitwagen C.L.J.J., Peters A.C.B., Jennekens-Schinkel A. (2003). Not only a matter of epilepsy: Early problems of cognition and behavior in children With “Epilepsy Only”-A prospective, Longitudinal controlled study starting at diagnosis. Pediatrics.

[21] Berg A.T., Hesdorffer D.C., Zelko F.A. (2011). Special education participation in children with epilepsy: what does it reflect?. Epilepsy Behav.

[22] Achenbach TM, Rescorla, L.A. Manual for the ASEBA School-Age Forms & Profiles. Burlington, VT: University of Vermont, Research Center for Children, Youth & Families; 2001.

[23] Official Statistics of Finland (OSF): Support for learning [e-publication]. http://www.stat.fi/til/erop/2012/erop_2012_2013-06-12_tau_001_en.html. 2012 [Accessed 30 October 2024].

[24] Lystad R.P., McMaugh A., Herkes G., Badgery-Parker T., Cameron C.M., Mitchell R.J. (2022). The impact of childhood epilepsy on academic performance: A population-based matched cohort study. Seizure: European. J Epilepsy.

[25] Jones C., Atkinson P., Helen Cross J., Reilly C. (2018). Knowledge of and attitudes towards epilepsy among teachers: A systematic review. Epilepsy Behav.

